# Type-1 cannabinoid receptors colocalize with caveolin-1 in neuronal cells

**DOI:** 10.1016/j.neuropharm.2007.06.030

**Published:** 2008-01

**Authors:** Monica Bari, Sergio Oddi, Chiara De Simone, Paola Spagnolo, Valeria Gasperi, Natalia Battista, Diego Centonze, Mauro Maccarrone

**Affiliations:** a Department of Experimental Medicine and Biochemical Sciences, University of Rome ‘Tor Vergata’, 00133 Rome, Italy; b European Center for Brain Research (CERC)/IRCCS, S. Lucia Foundation, 00196 Rome, Italy; c Department of Biomedical Sciences, University of Teramo, 64100 Teramo, Italy; d Neurologic Clinics, Department of Neurosciences, University of Rome ‘Tor Vergata’, 00133 Rome, Italy

**Keywords:** Caveolin, Cholesterol, Endocannabinoids, G protein-coupled receptors, Lipid rafts, Neurodegeneration, Signal transduction, Trafficking, AEA, anandamide (arachidonoylethanolamide), 2-AG, 2-arachidonoylglycerol, CAV1, caveolin-1, CB1/2R, type 1/2 cannabinoid receptor, DAGL, diacylglycerol lipase, FAAH, fatty acid amide hydrolase, GAR-AP, goat anti-rabbit alkaline phosphatase conjugate, GPCR, G protein-coupled receptor, IK, Ikaros, MCD, methyl-β-cyclodextrin, MAGL, monoacylglycerol lipase, NAPE, *N*-acyl-phosphatidylethanolamine, PLD, phospholipase D, TRPV1, transient receptor potential channel vanilloid receptor subunit 1

## Abstract

Type-1 (CB1) and type-2 (CB2) cannabinoid receptors belong to the rhodopsin family of G protein-coupled receptors, and are activated by endogenous lipids termed “endocannabinoids”. Recent reports have demonstrated that CB1R, unlike CB2R and other receptors and metabolic enzymes of endocannabinoids, functions in the context of lipid rafts, i.e. plasma membrane microdomains which may be important in modulating signal transduction. Here, we present novel data based on cell subfractionation, immunoprecipitation and confocal microscopy studies, that show that in C6 cells CB1R co-localizes almost entirely with caveolin-1. We also show that trafficking of CB1R in response to the raft disruptor methyl-β-cyclodextrin (MCD) is superimposable on that of caveolin-1, and that MCD treatment increases the accessibility of CB1R to its specific antibodies. These findings may be relevant for the manifold CB1R-dependent activities of endocannabinoids, like the regulation of apoptosis and of neurodegenerative diseases.

## 1 Introduction

Anandamide (AEA) and 2-arachidonoylglycerol (2-AG) are the most prominent members of “endocannabinoids” (Bari et al., 2006a; Di Marzo, 2006). They bind to and activate two inhibitory G protein-coupled receptors (GPCR), namely type-1 (CB1R) and type-2 (CB2R) cannabinoid receptors (Howlett, 2005). CB1R is localized mainly in the central nervous system (Egertova et al., 2003), but also expressed in peripheral tissues like immune cells (Klein et al., 2003). Conversely, CB2R is predominantly expressed peripherally, but it is also present in the brain (Nunez et al., 2004; Van Sickle et al., 2005). Activation of CB1 or CB2 receptors by AEA or 2-AG has many central and peripheral effects (Bari et al., 2006a; Di Marzo, 2006), obtained by triggering common signaling pathways (Howlett, 2005; Di Marzo, 2006). It would be of utmost importance to identify a possible differential regulation of CB1R and CB2R, also in view of the fact that these two receptor subtypes have been recognized as distinct drug discovery targets for numerous potential therapeutic applications; these include food intake, cancer and immune suppression (Bifulco and Di Marzo, 2002; Piomelli, 2003; Fowler, 2005). CB receptors, together with the enzymes that synthesize (*N*-acyl-phosphatidylethanolamines-hydrolyzing phospholipase D, NAPE-PLD) (Okamoto et al., 2004) or degrade (fatty acid amide hydrolase, FAAH) (McKinney and Cravatt, 2005) AEA, the enzymes that synthesize (diacylglycerol lipase, DAGL) (Bisogno et al., 2003) or hydrolyze (monoacylglycerol lipase, MAGL) (Dinh et al., 2002) 2-AG, and the AEA-binding transient receptor potential channel vanilloid receptor subunit 1 (TRPV1), form the “endocannabinoid system” (Piomelli, 2003; Bari et al., 2006a; Di Marzo, 2006).

Lipid rafts are subdomains of the plasma membrane that contain high concentrations of cholesterol and glycosphingolipids, and are well-known modulators of GPCR-dependent signaling and membrane trafficking in central and peripheral cells (Gajate and Mollinedo, 2005; Rodgers et al., 2005). Not surprisingly, they had been proposed also as potential regulators of CBR activity (Hinz et al., 2004; Barnett-Norris et al., 2005; McFarland and Barker, 2005). Recently, we have shown that treatment of rat C6 glioma cells for 30 min with the lipid rafts disruptor methyl-β-cyclodextrin (MCD) doubles the binding efficiency (i.e., the maximum binding:affinity constant ratio) of CB1R, and thus the CB1R-dependent actions of endocannabinoids; instead, MDC did not affect those activities mediated by CB2R or TRPV1 (Bari et al., 2005). In addition, we found that the activity of the endocannabinoid-metabolizing enzymes NAPE-PLD, FAAH, DAGL and MAGL is not affected by MCD, neither in central nor in peripheral cells (Bari et al., 2006b). In this context, it should be recalled that caveolae are a specialized subclass of lipid rafts, involved in cholesterol trafficking within most cell types, endocytosis of external molecules, and regulation of several signal transduction pathways (Arcangeli and Becchetti, 2006). They have been proposed as platforms for the accumulation of signaling molecules, and may provide a physical location for the initiation of downstream signaling events (Smythe et al., 2003). Caveolae have a characteristic protein coat, a major component of which is caveolin (Sotgia et al., 2006). To date three major caveolin isoforms have been identified, and the most widespread of them, caveolin-1 (CAV1), is expressed also in C6 cells (Silva et al., 1999).

In order to ascertain whether CB1R could reside within caveolae, here we investigated the colocalization of CB1R with CAV1 in C6 cells, by using subfractionation, immunoprecipitation and confocal microscopy techniques. We also investigated the trafficking of CB1R and CAV1 in response to MCD treatment, in the light of its potential relevance for the regulation of apoptosis and neurodegenerative diseases.

## 2 Materials and methods

### 2.1 Antibodies

Rabbit anti-CB1R polyclonal antibodies were from Affinity BioReagents, Inc. (Golden, CO). Rabbit anti-CAV1, anti-TRPV1, and anti-CAV1 mouse monoclonal antibodies were from Santa Cruz Biotechnologies (Santa Cruz, CA). Anti-NAPE-PLD rabbit polyclonal antibodies were from Cayman Chemical (Ann Arbor, MI). Anti-FAAH polyclonal antibodies were elicited in rabbits against the conserved FAAH sequence VGYYETDNYTMPSPAMR (Giang and Cravatt, 1997) conjugated to ovalbumin, and were prepared by Primm S.r.l. (Milan, Italy). Anti-thyroid hormone receptor mouse monoclonal antibody was from Calbiochem (La Jolla, CA). Goat anti-rabbit alkaline phosphatase conjugates (GAR-AP) were from Bio-Rad (Hercules, CA).

### 2.2 Cell culture and treatment

Rat C6 glioma cells were cultured in RPMI 1640 medium (Invitrogen Co., Carlsbad, CA), supplemented with 10% fetal bovine serum. Cells were maintained at 37 °C in a humidified atmosphere with 5% CO_2_ and were fed every 2 days. Lipid raft disruption was performed by preincubating C6 cells for the indicated periods of time at 37 °C with 2.5 mM MCD (Sigma Chemical Co., St. Louis, MO), followed by a washing step in phosphate-buffered saline (Bari et al., 2005).

### 2.3 Purification of caveolae-enriched membrane fractions

Fractions enriched in caveolae were prepared by carbonate extraction followed by gradient centrifugation (Song et al., 1996), adapted to C6 cells as reported (Silva et al., 1999). Homogenates from C6 cells (300 × 10^6^ cells/test) were placed at the bottom of a discontinuous sucrose density gradient and were spun in an SW41 Ti rotor (Beckman Instruments, Palo Alto, CA) at 188,000 × *g* and 4 °C for 22 h. Twelve 1 ml fractions were collected from top to bottom, saving also the heavy pellet. Proteins from each fraction were precipitated with 7.2% (v/v) trichloroacetic acid, and were solubilized in SDS–PAGE sample buffer (Xiang et al., 2002). The precipitated proteins (50 μg/lane) were separated on 12% SDS–PAGE gel, were electrotransferred on 0.45 μm nitrocellulose filters (Bio-Rad), and then were subjected to Western blot with rabbit anti-CAV1 (diluted 1:250) or rabbit anti-CB1R (1:250) specific antibodies.

### 2.4 Immunoprecipitation

C6 cells (100 × 10^6^/test) were sonicated in immunoprecipitation buffer (10 mM Tris–Cl, pH 8, 150 mM NaCl, 2 mM phenylmethanesulfonyl fluoride, 60 mM octyl glucoside) and centrifuged at 4 °C for 15 min at maximal speed in a microcentrifuge, and extracts were prepared as reported (Rybin et al., 2000). Immunoprecipitation was performed with the Protein G immuprecipitation kit from Sigma Chemical Co. (St. Louis, MO) according to the manufacturer's instructions. Briefly, whole cell extracts (1 mg) were incubated overnight at 4 °C with monoclonal antibody anti-caveolin1 (5 μg) or with an irrelevant monoclonal antibody anti-thyroid hormone receptor (5 μg), then immunocomplexes were incubated with 30 μl of protein G-Sepharose beads for 2 h at 4 °C. Beads were washed three times with immunoprecipitation buffer, then bound proteins were eluted with 100 μl of SDS–PAGE sample buffer and boiled for 5 min (Rybin et al., 2000). Cell lysate (20 μg), immunodeplete supernatants (20 μg/lane) and immunoprecipitates (4 μl/lane, corresponding to 4% of the immunoprecipitate) were immunoblotted with rabbit polyclonal antibodies against caveolin-1 (1:250), CB1R (1:250), NAPE-PLD (1:250), FAAH (1:250) and TRPV1 (1:250).

### 2.5 Confocal microscopy

C6 cells were settled on a glass coverslip at a density of 2 × 10^4^ cells/cm^2^. After 24 h C6 cells were treated with 2.5 mM methyl-β-cyclodextrin for the indicated time or left untreated (control cells). Cells were then fixed for 10 min at room temperature with 4% paraformaldehyde and processed for immunofluorescence (Terrinoni et al., 2004). Rabbit anti-CB1R antibodies (diluted 1:100), and mouse anti-CAV1 antibodies (1:100) were made fluorescent by using the “Alexa Fluor 488 and 546 Monoclonal Antibody Labeling Kit” (Molecular Probes, Eugene, OR). After immunofluorescence, coverslips were mounted using Prolong Antifade Kit (Molecular Probes), and data were acquired through a C1 confocal microscope (Nikon Instruments S.p.A., Florence, Italy) at an excitation of 488 nm (band of Ar laser) or 546 nm (band of a HeNe laser).

## 3 Results

### 3.1 Colocalization of CB1R and CAV1

**Fig. 1:**
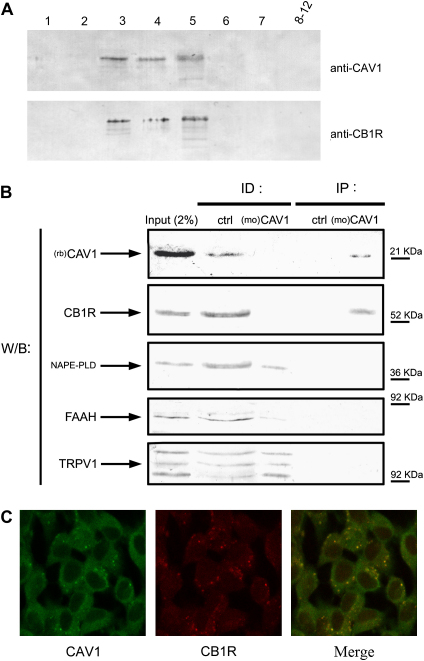
Localization of CB1R in C6 cells. (A) Western blot analysis of C6 cell membranes (50 μg/lane) from gradient fractions stained for caveolin-1 (anti-CAV1) or CB1 receptor (anti-CB1R). (B) Western blot analysis of C6 cell lysates immunoprecipitated with caveolin-1 specific IgG1 ((mo)CAV1), or irrelevant anti-thyroid hormone receptor antibody (ctrl), and subjected to SDS–PAGE. Immunoblot analysis of whole cell extract (input), immunodepleted supernatants (ID) and immunoprecipitates (IP) was performed using rabbit polyclonal antibodies against CAV1 ((rb)CAV1), CB1R, NAPE-PLD, FAAH and TRPV1. Molecular weights of marker proteins are indicated on the right. C, Colocalization of CAV1 and CB1R by confocal microscopy. The yellow spots indicate that CAV1 and CB1R colocalize, and are present mainly in intracellular vesicles. Membrane fractionation, immunoprecipitation, confocal microscopy and dilution of specific antibodies were as described in Section 2. Reported blots and images are representative of triplicate experiments.

Membrane fractionation studies were carried out to access the distribution of CB1R and CAV1 in C6 cells. CB1R was found exclusively in membrane fractions enriched with CAV1 (Fig. 1A), and its targeting to caveolae was further corroborated by immunoprecipitation studies. The latter showed that almost all CB1R co-immunoprecipitated with CAV1 in C6 cell extracts as revealed by the complete immunodepletion of CB1R in the flowthrough of the immunoprecipitation (Fig. 1B). Instead TRPV1, NAPE-PLD and FAAH did not co-immunoprecipitate with CAV1 (Fig. 1B), in keeping with previous biochemical data showing that they do not function within lipid rafts (Bari et al., 2005, 2006b). On the other hand, the lack of commercially available antibodies specific for DAGL or MAGL did not allow to further extend to these proteins the co-localization studies.

Additional confocal microscopy analysis further supported the colocalization of CB1R and CAV1 in C6 cells, demonstrating that CB1 receptors are predominantly localized intracellularly (Fig. 1C). It should be recalled that recent data in HEK-293 cells transfected with CB1R-EGFP (enhanced green fluorescence protein) plasmids have shown that a substantial proportion (≈85%) of receptors is indeed localized in intracellular vesicles (Leterrier et al., 2004).

### 3.2 Trafficking of CB1R and CAV1 upon MCD treatment

**Fig. 2:**
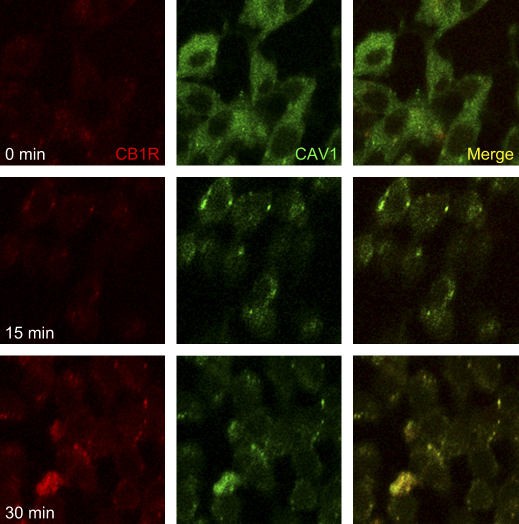
Trafficking of CAV1 and CB1R upon MCD treatment. C6 cells were treated for the indicated periods of time with 2.5 mM MCD, and immunofluorescence was recorded at 40× magnification. Cells were double stained with antibodies against CB1 receptors (red) and caveolin-1 (green). Reported images are representative of triplicate experiments.

**Fig. 3:**
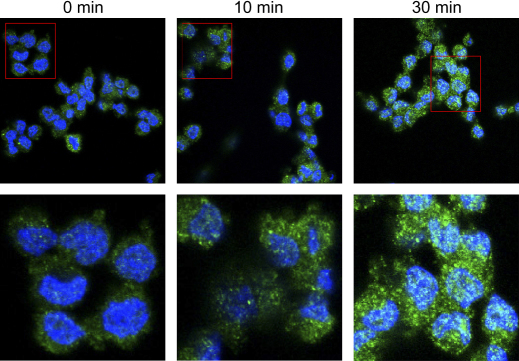
Effect of MCD on CB1R immunoreactivity. C6 cells were treated for the indicated periods of time with 2.5 mM MCD, and immunofluorescence was recorded at 20× (upper panels) or at 60× (lower panels) magnification. Cell nuclei were stained in blue with DAPI, whereas localization of CB1 receptors is shown as green spots. Reported images are representative of triplicate experiments.

CB1R has been shown to permanently and constitutively cycle between plasma membrane and endosomes (Hsieh et al., 1999; Leterrier et al., 2004). Therefore, we sought to ascertain the possible role of CAV1 in this trafficking. To this end, we examined the localization patterns of CB1R and CAV1 in C6 cells treated with MCD, that disrupts raft integrity in C6 cells (Bari et al., 2005) as well as in many other cell types (Rybin et al., 2000: Kunzelmann-Marche et al., 2002; Xiang et al., 2002). MCD caused a time-dependent recycling of CB1R, that was superimposable on that of CAV1 (Fig. 2). Furthermore, MCD led to a time-dependent increase of CB1R immunoreactivity (Fig. 3), at the same dose (2.5 mM) and in the same time range (0–30 min) that has been shown to double the number of binding sites of the receptor, and hence its binding efficiency (Bari et al., 2005). It seems noteworthy that this time frame is not compatible with de novo protein synthesis, suggesting that MCD treatment might increase the accessibility of the binding sites to the anti-CB1R antibodies. Interestingly, these antibodies have been shown to antagonize the effect of natural or synthetic CB1R agonists (Maccarrone et al., 2005), suggesting that indeed they react with the binding site of the receptor.

## 4 Discussion

Lipid rafts are subdomains of the plasma membrane that contain high concentrations of cholesterol and glycosphingolipids, and are well-known modulators of GPCR-dependent signaling and membrane trafficking in central and peripheral cells (Gajate and Mollinedo, 2005; Rodgers et al., 2005).

We have demonstrated that, in rat C6 glioma cells, raft perturbation by MCD treatment enhances CB1R binding and signaling (Bari et al., 2005). Shortly afterwards, independent reports have shown that CB1R is localized within lipid rafts also in human MDA-MB231 cells, a breast cancer cell line (Sarnataro et al., 2005, 2006), and in human endothelial cells (Bari et al., 2006b). Remarkably, the different dependence of CB1 and CB2 receptors on raft integrity was observed also in primary cells (Bari et al., 2006b).

Here, we supplement novel information that demonstrates that CB1R resides almost entirely in a specialized type of lipid rafts, the caveolae. This observation suggests a strong link between CB1R and CAV1, that seems interesting because caveolae play a role in neurodegenerative diseases, like Parkinson's disease, Alzheimer's disease and dementia with Lewy's bodies (Hashimoto et al., 2003), as well as in other neurological abnormalities (Trushina et al., 2006). Notably, also endocannabinoids have been shown to interfere with these processes through CB1R-dependent mechanisms (Van der Stelt and Di Marzo, 2005; Maccarrone et al., 2007).

Caveolae represent a subset of lipid rafts, characterized by high caveolin content and formation of 50–100 nm flask-shaped membrane invaginations (Pike, 2003). Caveolins are a family of scaffolding proteins critical for the organization of preassembled signaling complexes within the plasma membrane, and are known to regulate the activity of several G proteins (Okamoto et al., 1998; Simons and Toomre, 2000; Simons and Ehehalt, 2002; Pike, 2003). The CAV1 isoform has been identified in C6 cells (Silva et al., 1999), as well as in many other rat and human glioma cells (Cameron et al., 2002). Our present finding that CB1R co-localizes with CAV1 in this type of cells (Fig. 1A–C) suggests that at least part of the enhancement of CB1R signaling observed upon lipid raft disruption by MCD (Bari et al., 2005) may involve CAV1. In support of this hypothesis, we show that MCD causes a CB1R trafficking that is superimposable on that of CAV1 (Fig. 2), and that it enhances the accessibility of receptor binding sites to specific anti-CB1R antibodies (Fig. 3). Of particular relevance seems the fact that activation of CB1R protects neurons, astrocytes and several peripheral cells against apoptosis (Guzman, 2003; Maccarrone, 2006). In line with this, lipid rafts disruption has been shown to block apoptosis induced in vitro by AEA in glioma cells (Sarker and Maruyama, 2003) and hepatocytes (Biswas et al., 2003), as a consequence of CB1R activation (Bari et al., 2005). On the other hand activation of CB1R can also promote apoptosis in vitro, for instance in glioma cells (Galve-Roperh et al., 2000), and in rat cortical astrocytes and human astrocytoma cells (Sànchez et al., 2001). Generally speaking, it seems that activation of CB1 receptors modulates the balance among different signals (like FAN, ERK, JNK and p38 MAPK, PI3K/PKB), that in turn impact the cell choice between proliferation and death, driving it towards apoptotic or anti-apoptotic pathways (reviewed by Maccarrone, 2006). At any rate, CB1R-dependent pro-apoptotic signals may contribute to the pro-apoptotic activity of CAV1. In fact this substance, befitting its role as a multi-tasking molecule, has been shown to sensitize cells towards apoptosis, by regulating cell cycle progression and by activating pro-apoptotic signals like bcl2, p53 and p21 (Shajahan et al., 2007). Since several human glioblastoma tumors possess CAV1 (Cameron et al., 2002), and depletion of cholesterol in lipid rafts has been shown to inhibit apoptosis induced by anti-tumor drugs (Gajate and Mollinedo, 2001), this investigation raises the suggestive although still speculative idea that perturbation of lipid rafts, by modulating CB1R- and CAV1-dependent signaling, may be involved in finely tuning cell survival and death within the central nervous system. This concept might be exploited for the treatment of endocannabinoid-related diseases that are CB1R-dependent, such as brain injury (Mechoulam et al., 2002) and oxidative stress (Carracedo et al., 2004), cancer (Guzman, 2003), and neurodegenerative disorders (Piomelli, 2003; Maccarrone et al., 2007).
